# Exploring long-range proton conduction, the conduction mechanism and inner hydration state of protein biopolymers†

**DOI:** 10.1039/c9sc04392f

**Published:** 2020-03-11

**Authors:** Somen Mondal, Yuval Agam, Ramesh Nandi, Nadav Amdursky

**Affiliations:** Schulich Faculty of Chemistry, Technion – Israel Institute of Technology Haifa 3200003 Israel amdursky@technion.ac.il

## Abstract

Proteins are the main proton mediators in various biological proton circuits. Using proteins for the formation of long-range proton conductors is offering a bioinspired approach for proton conductive polymers. One of the main challenges in the field of proton conductors is to explore the local environment within the polymers, along with deciphering the conduction mechanism. Here, we show that the protonic conductivity across a protein-based biopolymer can be hindered using straightforward chemical modifications, targeting carboxylate- or amine-terminated residues of the protein, as well as exploring the effect of surface hydrophobicity on proton conduction. We further use the natural tryptophan residue as a local fluorescent probe for the inner local hydration state of the protein surface and its tendency to form hydrogen bonds with nearby water molecules, along with the dynamicity of the process. Our electrical and spectroscopic measurements of the different chemically-modified protein materials as well as the material at different water–aprotic solvent mixtures result in our fundamental understanding of the proton mediators within the material and gaining important insights on the proton conduction mechanism. Our biopolymer can be used as an attractive platform for the study of bio-related protonic circuits as well as a proton conducting biopolymer for various applications, such as protonic transistors, ionic transducers and fuel cells.

## Abstract 2

Post formation modification of protein-based materials can attenuate the proton conduction efficiency resulting from change in conduction mechanism, charge carrier mobility, carrier concentrations and inner hydration layer.

## Graphical abstract

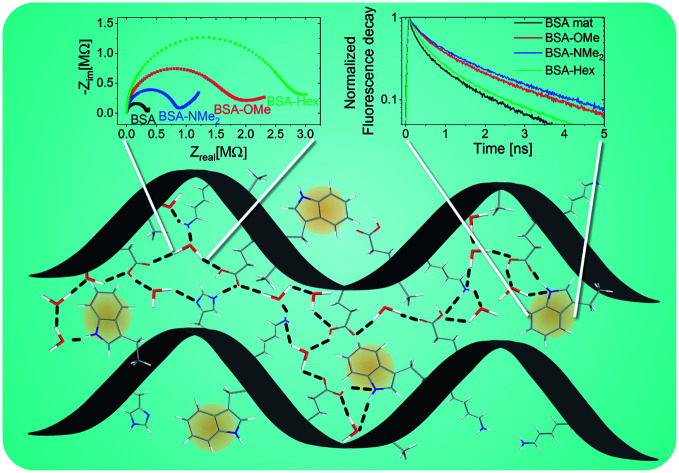

## Introduction

Directional proton transport (PT) is one of the most fundamental processes in biology, which takes place within proteins and usually across the two sides of a membrane, such as in light- or voltage-activated channels or the protein complexes participating in photosynthesis and aerobic respiration.^1–5^ Several proton pathways in natural proton mediating proteins have been identified, all involving structural water molecules and specific amino acids (mostly the oxo-amino acids of Glu and Asp) participating in the formation of a hydrogen bond network spanning in the direction of the PT pathway. The ability of proteins to support short-range (nm-scaled) PT in nature has resulted in several recent works showing the ability of proteins to support macroscopic proton conduction (PC).^6–9^ As shown by Ordinario *et al.*, one way to manipulate PC across the protein material is by mutagenesis, where the authors replaced all the Glu and Asp in the protein to alanine. This mutagenesis resulted in a dramatic order of magnitude decrease in measured current density, and further proved the importance of these amino acids in supporting long-range PC. Nevertheless, mutagenesis is not a straight-forward approach, requiring molecular biology tools, and it is not accessible to most materials groups.^8^ The importance of specific amino acids in long range PC has also been demonstrated by the use of self-assembled peptides, where Silberbush *et al.* showed that Glu containing peptide is more conducting than Lys containing peptide, in which both are more conducting than Gln containing peptide.^10^ In here, we use bovine serum albumin (BSA) electrospun mats as a solely proton-conducting (*i.e.*, no heavier ions) protein biopolymer.^7,11^ The choice of BSA is due to its ability to form macroscopic free-standing (self-supporting) material, a property that is unavailable for ‘common’ proton mediating proteins. We show our ability to hinder the protonic conductivity using facile chemical modifications, targeting carboxylate containing amino acids (Glu and Asp) or primary amine containing ones (Lys) within the protein biopolymer. Our modifications include functionalizing with methylester, *N*-methylamine and *N*-hexylamide, and they significantly change the inner hydration state of the material, meaning the level of hydration surrounding the residues of the BSA protein surface within the mat. This ‘inner’ surface is referring to the surface of the BSA protein fibrils comprising the mat that is exposed to water (or other solvent). Due to the importance of water molecules in facilitating PC across any proton conducting material, this inner hydration state has a major role in the PC efficiency. While estimating the water uptake of the material is rather simple, following the inner hydration layer, *i.e.*, the close water layer around the material itself, is a complicated task. Here, we use the natural fluorescent amino acid of Trp as our local probe, in which its spectroscopic characteristics are highly sensitive to its local environment.^12–15^ Using time-resolved measurements has further allowed us to isolate specific solvation dynamics surrounding the Trp, the formation of hydrogen-bonds and determine the structural flexibility. Our study pinpoints the importance of natural oxo-amino-acids and amine groups as proton-hopping sites as well as their interactions with water molecules in supporting long-range PC across proteins.

## Results

**Scheme 1:**
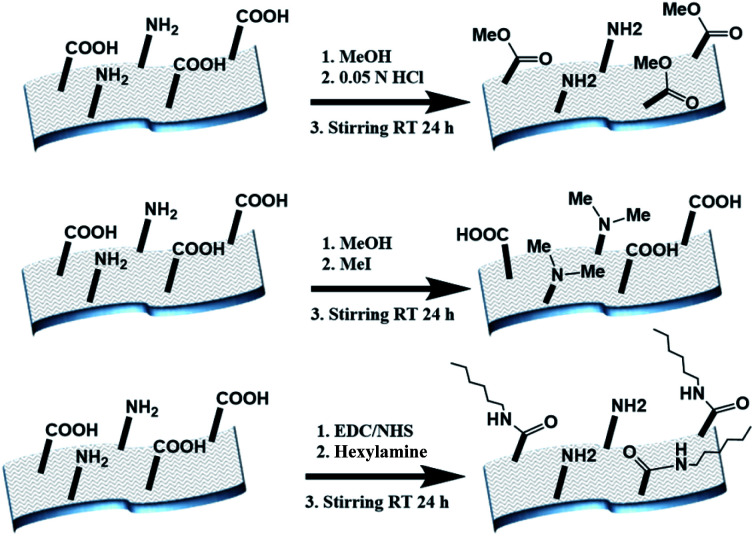
The different chemical modifications of the BSA mat used in this study, from top to bottom: BSA-OMe, BSA-NMe_2_ and BSA-Hex.

The electrospun BSA mats were chemically modified targeting different amino acids with different modifications (Scheme 1 and further details in the experimental section). For exploring the role in the PC of carboxylic acids containing residues (Glu and Asp) *vs.* primary amine containing residues (Lys), we have functionalized the BSA mat with methyl ester (BSA-OMe) or *N*-methylation (BSA-NMe_2_) modifications, respectively. To explore the role of the surface hydrophobicity in PC, we have functionalized Glu and Asp with long chain hexylamine (BSA-Hex). Following the chemical modifications, the modified BSA mats were characterized by FTIR and Raman spectroscopies (Fig. S1 and S2,† along with a discussion within).

**Fig. 1:**
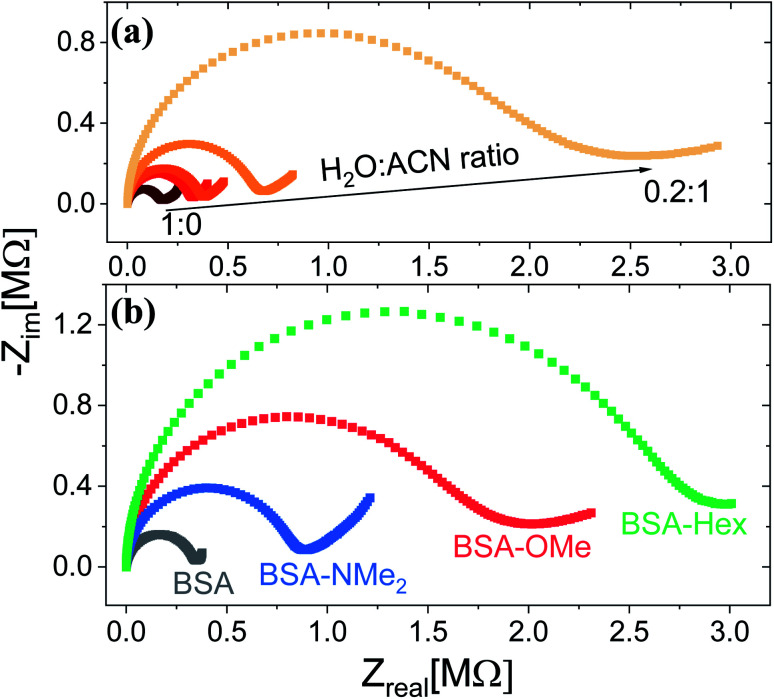
Proton conduction across the BSA mat as measured with EIS at (a) different water : ACN mixture, and upon (b) different chemically-functionalized BSA mats.

Following the chemical characterization of the different BSA mats, we followed the PC across them. In here, we have used two strategies in order to explore the role of the inner hydration layer within the BSA mat on its ability to support long-range (2.5 mm) PC. The first is to immerse the mat in different ratio of aprotic solvent, while the second is to use our modified mats with different degree of inner surface hydrophobicity. It is expected that with increasing ratio in favour of the aprotic solvent (acetonitrile (ACN) in our case), the protonic conductivity across the BSA mat will be diminished due to lesser availability of water molecule *i.e.* less proton source and poor H-bonds network. Indeed, as shown in Fig. 1a, the protonic conductivity (as measured with electrochemical impedance spectroscopy (EIS) in the form of a Nyquist plot) is highly sensitive to the water–ACN ratio within the BSA mat, in which we observed a striking order of magnitude difference between the fully hydrated BSA mat to the one containing 20% water (water : ACN ratio of 0.2 : 1). Our first strategy already confirmed the crucial role of water molecules in supporting PC across the BSA mat, but for gaining a fundamental understanding of the protein side groups in the conduction mechanism, we used our chemically modified BSA mats. As shown in Fig. 1b, we observed a distinct effect of the different functional group modification on the protonic conduction, in which the conductivity of the native unmodified BSA mat was significantly higher than all other modified BSA mats. It is important to note in this stage that all the different BSA mats were hydrated with around similar amount of water: ∼80 wt% for BSA, BSA-OMe and BSA-NMe_2_ and ∼73 wt% for BSA-Hex, in which the slightly lower amount of water in the latter is probably due to the more hydrophobic nature of the BSA-Hex mat. We observed that different modifications resulted in different conductivity values (Table 1), whereas the BSA-NMe_2_, BSA-OMe and BSA-Hex are ∼2.9, 5.3 and 8.3 folds less conducting compared to the unmodified BSA mat. The first important conclusion of this stage is that blocking free carboxylates from forming H-bonds by methylation (BSA-OMe) is more crucial for efficient PC than blocking free primary amines from forming H-bonds by methylation (BSA-NMe_2_). This conclusion implies that oxo-amino acids are more important in transporting protons across proteins compared to primary amines, even though both can contribute to the integrity of the H-bonds network and both can be protonated. The second important conclusion is that modifying free carboxylates with 6-carbon aliphatic chain (BSA-Hex) resulted in a less conductive mat than the modification with a single carbon methylation (BSA-OMe). This conclusion implies significant changes in the local hydration layer around the surface of the BSA fibrils within the mat between BSA-OMe to the more hydrophobic BSA-Hex.

**Table 1:** Calculated conductivity as measured by EIS, and mobility and carrier density values, as measured by FET, for the different chemical modified BSA mat

Sample	Conductivity (S cm^−1^)	Mobility (cm^2^ V^−1^ S^−1^)	Carrier density (cm^−3^)
BSA-mat	1.02 ± 0.31 × 10^−4^	6.1 ± 0.08 × 10^−3^	1.05 × 10^17^
BSA-OMe	1.93 ± 0.18 × 10^−5^	3.3 ± 0.08 × 10^−3^	3.66 × 10^16^
BSA-NMe_2_	3.50 ± 0.06 × 10^−5^	4.3 ± 0.10 × 10^−3^	5.09 × 10^16^
BSA-Hex	1.23 ± 0.18 × 10^−5^	1.9 ± 0.12 × 10^−3^	4.05 × 10^16^

**Fig. 2:**
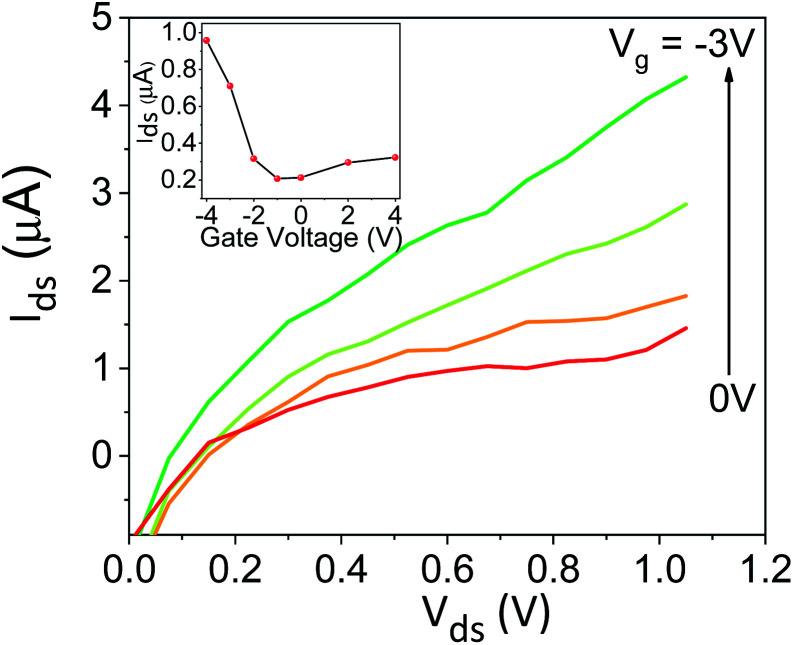
Protonic FET measurement, source–drain current (*I*_DS_) as a function of the source–drain voltage (*V*_DS_) at different values of gate voltage *V*_GS_ for unmodified BSA mat. The inset shows the transfer line characteristics of the device.

To explore the protonic mobility (*μ*) and carrier density (*n*) for the different chemically-modified BSA mats, we switched to protonic field effect transistor (FET) devices. Here, we measured the source drain current (*I*_DS_) against the drain–source bias (*V*_DS_) while modulating the gate voltage (*V*_GS_) (Fig. 2 and S3†). The transfer characteristic (inset Fig. 2) exhibits higher *I*_DS_ current magnitude at negative gate voltages, which is indicative for p-type carriers, where in our devices those are the positive protons. We have used our measurements (at the low negative gate bias regime) to calculate the protonic mobility (*μ*) for the different BSA mat (Table 1) using the equation:
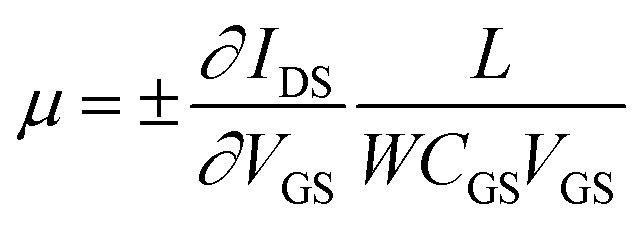
where, *L* is the channel length, *W* is the channel width and *C*_GS_ is the capacitance of the oxide between gate and source. After calculating the mobility values, we estimated the charge carrier density (*n*) for the different BSA mat (Table 1) using the basic conductivity equation: σ_H^+^_ = *μ*_H^+^_ + *n*_H^+^_ + *e*, where *σ*_H^+^_ is our measured proton conductivity and *e* is the elementary charge. Our calculated protonic mobility for the unmodified BSA mat is 6.1 × 10^−3^ cm^2^ V^−1^ S^−1^, which is in line with other reported mobility values for various biological materials.^8,16^ In accordance to our EIS measurements, we also calculated a decrease in the mobility values upon modifying the BSA mat, in which the mobility of BSA-NMe_2_, BSA-OMe and BSA-Hex are ∼1.4, 1.8 and 3.2 folds lower compared to the unmodified BSA mat. This is a further proof for the active role the amino acid side chains have in mediating PC. Interestingly, also the calculated charge carrier density is lower for all the different modification, even though the water content was rather similar for all samples (*vide supra*), in which for BSA-NMe_2_, BSA-OMe and BSA-Hex the values are ∼2.1, 2.9 and 2.6 folds lower compared to the unmodified BSA mat, respectively. This implies that the charges closest to the BSA fibrils within the mat are the important ones for our measured PC, supporting the role of the amino acids side chains in mediating protons. Hence, the more hydrophobic protein surface (*e.g.*, upon side chain modification), the less available charge carriers next to it. Thus, we can conclude that the lower conductivity of the modified BSA mats is resulted from both the lower protonic mobility and the lower charge carrier density.

In a short summary, our electrical measurements show that the surface modifications of the BSA mat results in significant changes in the PC efficiency that most probably associated with different PC mechanism across the mat. As stated, the hydration layer within the material has a crucial role in supporting long-range PC, and understanding the dynamics of this layer is of prime importance. This leads us to an inherent advantage of our BSA mats; they contain Trp, a fluorescent amino acid that is extremely sensitive to the polarity of its surrounding due to its ability (or inability) to form H-bonds between its indole ring and surrounding solvent molecules. Accordingly, Trp is frequently used as an intra-protein probe for the local environment of proteins or their level of denaturation.^12–15,17^

**Fig. 3:**
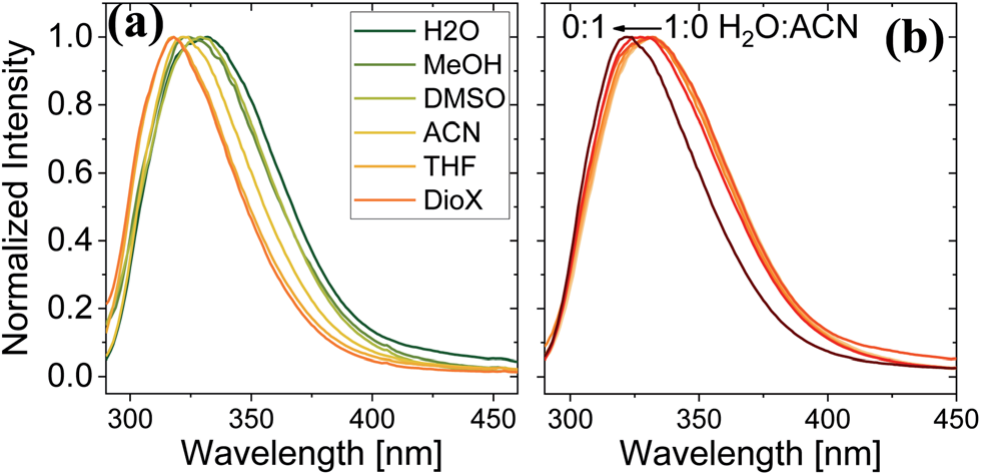
Normalized steady state fluorescence spectra of (a) BSA mat in different solvents, and (b) in different water : ACN mixtures.

Here, we use Trp as an intramolecular surface reporter to measure the local hydration state within the BSA mats, and specifically, next to the Trp residue. As will be discussed below, we found a correlation between the local hydration state next to the Trp residue to the measured PC across the entire mat. As above, we use different strategies to influence the hydration layer. The first strategy is to immerse the BSA mat in different solvents having different polarities and hydrogen bonding abilities. The steady-state fluorescence of the BSA mat with different solvents (Fig. 3a) clearly shows a shifting in the Trp emission peak position from 332 to 317 nm with decreasing solvent polarity from water all the way to dioxane. We further measure the Trp fluorescence at different water : ACN mixtures (Fig. 3b), and in accordance with the previous measurements, we observed a gradual shift in the emission peak position from 332 nm at pure water to 321 at pure ACN. Our results show that indeed Trp can be used as an inner probe within the BSA mat for the local hydration, and its fluorescence is related to whether it can form H-bonds (in protic solvents or high fraction of them) or not.

**Fig. 4:**
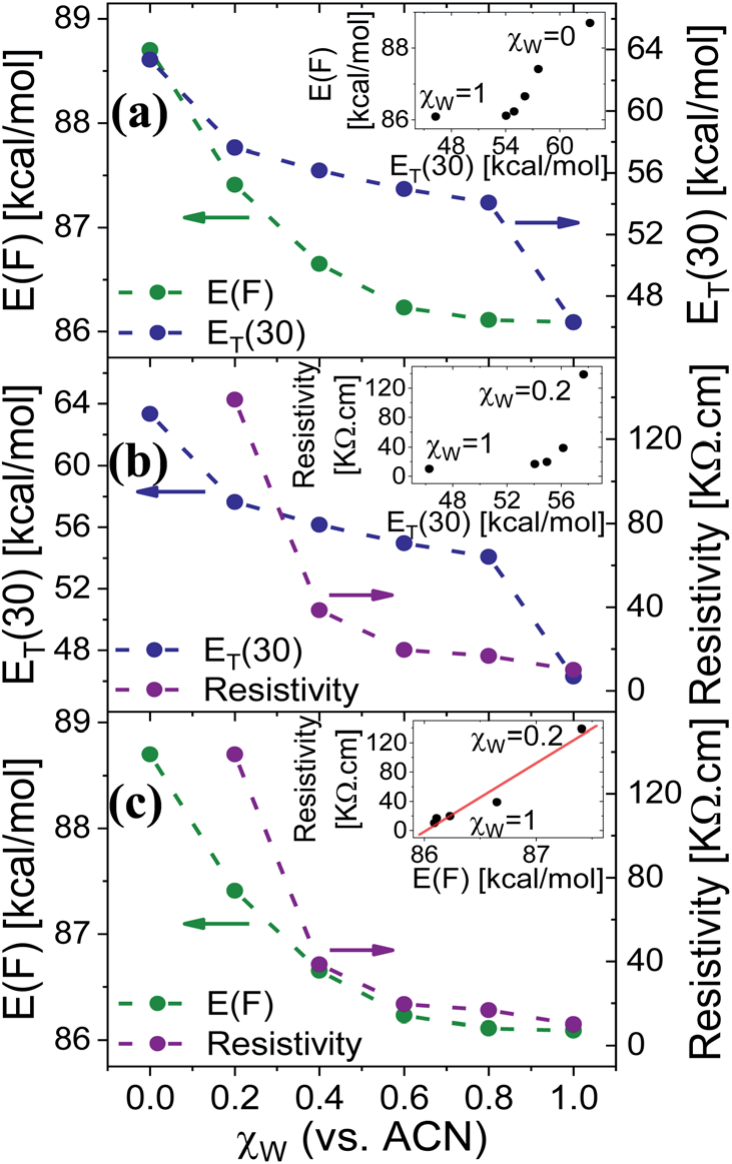
The dependence of (a) *E*(*F*) and *E*_T_(30), (b) *E*_T_(30) and resistivity, (c) *E*(*F*) and resistivity, as a function of the water fraction (within ACN), *χ*_w_, in the mat (for *E*(*F*) or resistivity) or in bulk solution (for *E*_T_(30)). The insets of (a and b) show the non-linear dependence of *E*(*F*) or resistivity *vs. E*_T_(30), and the one of (c) shows the linear dependence of the resistivity *vs. E*(*F*).

Measuring the Trp fluorescence at different water : ACN ratio is also allowing us to explore the differences (if any) between the interaction of the solvent with the surface to the one between the solvent molecules themselves (bulk solution). This can be done by comparing the Trp peak position energy (*E*(*F*) in units of kcal mol^−1^), which is indicative to solvent–surface interactions, to the common polarity parameter of *E*_T_(30), which is indicative of solvent–solvent interactions, for different mole fraction of water (*χ*_w_) in ACN.^18^ As can be seen in Fig. 4a, the change in *E*(*F*) is non-linear, while at *χ*_w_ > 0.6 it reaches nearly saturation, indicating the saturation of the Trp surrounding with water molecules together with its ability to form H-bonds with these water molecules. This non-linear behavior of *E*(*F*) is very different from the one of *E*_T_(30) that exhibits a large drop in values from neat ACN to *χ*_w_ = 0.2, a linear relation at 0.2 < *χ*_w_ < 0.8 and a large drop from *χ*_w_ = 0.8 to neat H_2_O. This large deviation between *E*(*F*) and *E*_T_(30) suggests that surface–solvent properties of the BSA mat are different from bulk solvent properties. The main question here is *whether the PC takes place along the surface of the BSA mat or through bulk solvent?* From Fig. 4b and c we can easily see that the resistivity of the BSA mats measured at different water : ACN ratio (Fig. 1a) follows the same behavior of *E*(*F*) and not the one of *E*_T_(30). This is a further strong indication to the involvement of the protein surface (and its amino acids side chains) in mediating the protons along the structure.

**Fig. 5:**
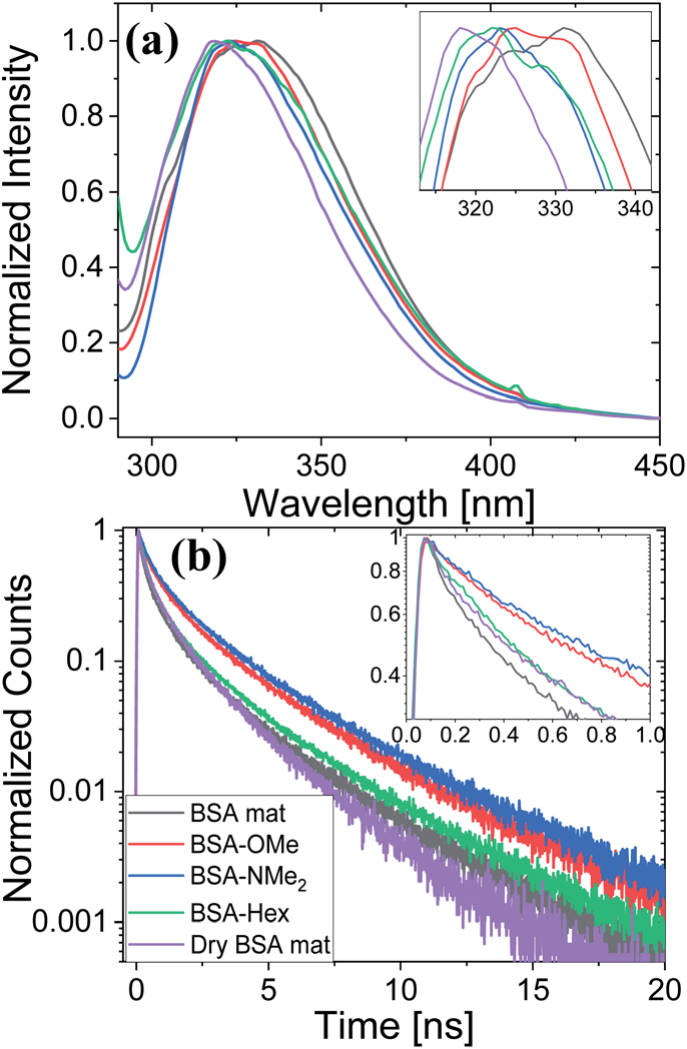
(a) Normalized steady state fluorescence spectra, and (b) time resolved fluorescence decay spectra of the different BSA mats used here compared to the native fluorescence of the BSA protein. The insets show a zoom-in of the Trp peak position and the first ns decay for (a) and (b), respectively.

Next, we have followed the Trp fluorescence of our various chemically modified BSA mats and compared them to the unmodified mat in its hydrated and dry forms. A dry BSA mat means the removal of all non-tightly bound water molecules, whereas the tightly bound water molecules constitutes around 7% of the dry BSA mat mass.^7^ As can be seen in Fig. 5a, the Trp emission peak position of the BSA mat undergoes significant hypsochromic shift upon drying the mat from 332 to 317 nm for the hydrated and dry BSA mats, respectively, similar to the location of the peak in aprotic solvents (Fig. 3a). The Trp emission peak positions of all the different BSA mats are located between the one of the hydrated BSA mat to the one of the dry mat. Noticeably, with increasing number of added (sp^3^) carbon modification we observed more hypsochromic shift, in the order of BSA-OMe (326 nm) → BSA-NMe_2_ (323 nm) → BSA-Hex (321 nm). Our results imply that the added hydrophobic interactions between the modified mat to the Trp residue induces a local dehydration in the vicinity of the aromatic residue.^19^

For exploring the time-domain dynamics of the inner hydration layer within the different BSA mats we turned into time-resolved fluorescence study of the Trp emission. The collected fluorescence decay traces (Fig. 5b) exhibit a bi-exponential nature (Table 2), one being in the ns time scale while the other in the sub-ns, which is commonly attributed to different Trp conformers.^20^ The fast decay component (*τ*_1_) can be ascribed to the solvation dynamics of the Trp with surrounding water molecules, whereas the slow decay component (*τ*_2_) can be ascribed to the less solvated Trp conformation due to its hydrophobic interaction with other moieties.^15^ Accordingly, a more solvated surrounding of the Trp will result in fast *τ*_1_ values along with its larger amplitude (*a*_1_) compared to the amplitude of the second component (*a*_2_). Indeed, Table 2 shows that while the fast component of the BSA mat contributes more than 54% of its decay, the one of the dry BSA mat is only 40%, and *τ*_1_ is faster for the hydrated mat (160 ps) compared to the dry mat (210 ps). The lifetimes values obtained with the BSA-Hex are very similar to the ones of the dry BSA mat, and we even received lower *a*_1_ for the BSA-Hex (30.2%) than the dry BSA mat, indicating the very dry environment surrounding the protein in this modification. Interestingly, we received rather similar amplitudes for BSA-OMe (43.3%) and BSA-NMe_2_ (42.4%) compared to the dry mat, albeit with much slower decay (*τ*_1_ = 300 and 320 ps for BSA-OMe and BSA-NMe_2_ respectively). These slower decays are probably due to local dehydration of the Trp only by the short methyl functionalization and not by the longer hexyl chain, in a rather similar trend as was reported for the interaction of Trp and urea derivatives.^19^

**Table 2:** Bi-exponential fitting^a^ of the Trp emission decay for the different BSA mats used here

Sample	*a*_1_ (%)	*τ*_1_ (ps)	*a*_2_ (%)	*τ*_2_ (ns)	〈*τ*〉^b^ (ns)
BSA mat-wet	54	160	46	1.35	0.74
BSA-OMe	43.3	300	56.7	2.10	1.33
BSA-NMe_2_	42.4	320	57.6	2.35	1.47
BSA-Hex	30.2	220	69.8	1.46	1.08
BSA mat-dry	40	210	60	1.45	0.95

a The “*a*” values are the relative amplitudes of each lifetime component. The *χ*^2^ values determine the goodness of the fits, which is >0.99 for all decays. Standard deviation for amplitude analysis is ±5%.

b Averaged lifetimes.

**Fig. 6:**
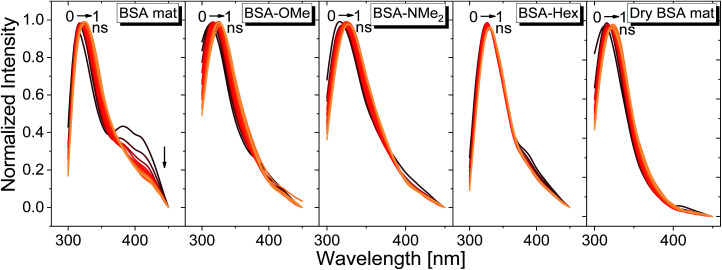
TRANES graphs of the different BSA mats used here for the first ns of Trp emission decay.

We have used our time-resolved measurements together with the steady-state ones to calculate the fast (1 ns) time-resolved area normalized emission spectra (TRANES) of the different BSA mats (Fig. 6). TRANES is a convenient tool to follow the emitting species of the excited state. We observed a gradual bathochromic shift as a function of time for all of the different BSA mats in their main emissive peak, which is due to the time-dependent solvation of the Trp. Interestingly, an additional very short-lived peak was found at ∼400 nm, which was most prominent at the hydrated BSA mat. We can ascribe this peak to a radical formation on the Trp, which was shown to undergo a proton coupled electron transfer process resulting in the formation of an hydronium ion *via* the cleavage of disulfide bond.^21,22^ Due to the importance of a water molecule as an acceptor for a proton in this process, it takes place mainly in the unmodified BSA mat. This intriguing process can be viewed as a UV-light-induced self-doping of the BSA mat with both electrons and ions, which might further contribute to the higher conductivity of the BSA mat in comparison to the different modification. It should be mentioned that melanin, a different biological material, has been also showed to produce free carriers (free radicals and hydronium ions) after absorption of water *via* comproportionation reaction, accountable for the high conductivity of melanin based materials.^23^ We have used the TRANES curves to calculate the hydration correlation function *C*(*t*) (Fig. S4†), *i.e.*, an exponential fitting for the solvation time constant, *C*(*t*), of the bathochromic shift, Δ*ν*, for the various BSA mats (Table 3). As expected, the solvation time constant for the hydrated mat (267 ps) is faster than the one of the dry mat (334 ps) with larger bathochromic shift. Surprisingly, the solvation time for all the various modification was even slower than the dry BSA mat, indicating the important role of the chemical modification on the ability of the Trp surrounding to be solvated. The slowest calculated time constants were for BSA-OMe (430 ps) and BSA-NMe_2_ (458 ps), which is in line with the slow Trp emission decay, and as discussed, is probably due to the specific interaction between the methyl group and the Trp indole ring that slows down the solvation time. BSA-Hex exhibit the smallest bathochromic shift (only 6 nm), which is a strong indication to the poor solvation taking place in this mat.

**Table 3:** The observed shift in the TRANES main emission peak along with the calculated solvation time constant

Sample	Δ*ν* (nm)	*C*(*t*) (ps)
BSA mat	17	267
BSA-OMe	16	430
BSA-NMe_2_	12	458
BSA-Hex	6	369
Dry BSA mat	12	334

## Discussion

In here, we have explored the role of the inner hydration layer in protein-based materials for their ability to support long-range PC. From a materials point of view, our protein-based material should be discussed in context of other biological materials that shown to support long range PC, such as films made by the reflectin protein, self-assembled peptides or polysaccharides.^8,16,26–28^ In the broader picture, all proton conducting biological materials should be compared to common ionic-conducting polymers (also known as ionomers), whereas Nafion® being the golden standard for efficient PC. Even though the PC values across our BSA mat is more than two orders of magnitude lower compared to the one of Nafion (0.1 and ∼50 mS cm^−1^, respectively), similar PC/PT mechanisms can take place. The common convention is that two different type of mechanisms can take place within ionomers.^24,25^ The first being proton diffusion within water channel inside the polymer, while the other involves proton hopping between water molecules and functional groups of the polymer (sulfonates in the case of Nafion). It is yet to be resolved whether proton diffusion within water channels is by hydronium ion movement in what is known as the vehicular mechanism (hydrodynamic transport) or *via* proton hopping steps across an H-bonds network spanning between water molecules within the channel in what is known as the Grotthuss mechanism (a prototropic mechanism).

**Scheme 2:**
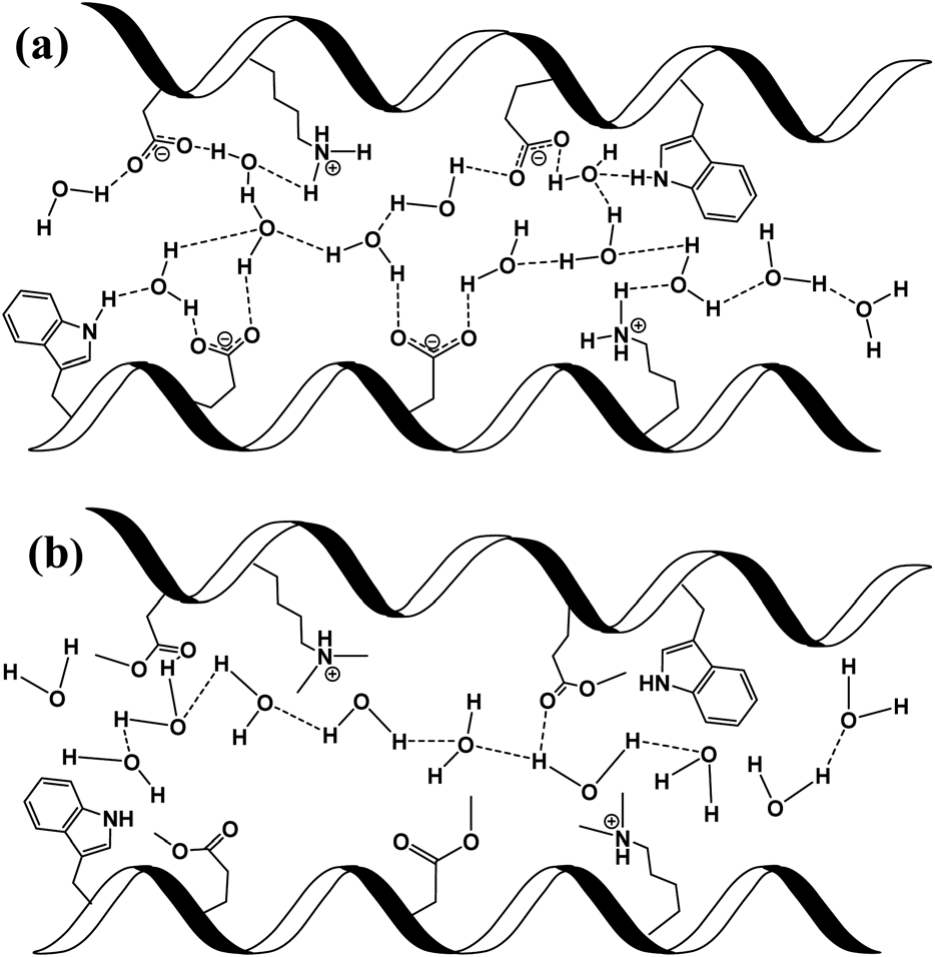
Schematic representation of individual PT steps and general PC pathway in (a) unmodified BSA mat, where protons are mediated *via* the formation of hydrogen bonds between amino acid side chains and water molecules; and (b) in modified BSA, where the hydrogen bond network is disrupted and protons are less interacting with the side groups of the protein.

In the next section, we will use our various measurements to discuss the contribution of the mentioned two conduction mechanisms of the native BSA mat *vs.* the different chemical modified mats (Scheme 2). The native BSA mat contains numerous protonable amino acids, and consequently it is more susceptible to form hydrogen bonds network with the water molecules within the mat (Scheme 2a). This situation is similar to other biological materials, such as the mentioned works with the reflectin protein or the polysaccharides.^8,16,26,27^ Accordingly, as suggested previously for the latter materials, we also suggest here that the PC across the native mat is due to proton hopping between water molecules and functional group (amino acid residues) on the surface of the BSA mat that can participate in an H-bond. In our current study, we show that the H-bonds network between water molecules and functional groups can be interrupted either by replacing water by an aprotic solvent or by blocking the latter mentioned amino acids using chemical modifications. The disruption of the H-bonds network in the various chemically-modified BSA mats can result in a situation where protons can diffuse along water channels with less interactions between water molecules and functional groups (Scheme 2b). Though it is important to mention that other amino acid as well as the amide bonds can also participate in the formation of H-bonds, hence we do not claim that in the chemically-modified BSA mat the mechanism is purely through water channels. The lack of any structural insight on the internal molecular organization within the BSA mat does not allow us to know to what degree the H-bonds network within the mat is disrupted upon the different chemical modifications.

To support our understanding of the PC mechanism across the various BSA mats in this study we turned to our results. We have used steady-state and time-resolved fluorescence study of the Trp residue to probe the local hydration layer within the various BSA mats, and showed that upon chemical modifications the surface hydration completely alters. More specifically, we showed that in the native mat, the Trp residue is more pronounced to form H-bonds with nearby water molecules than all other chemically-modified mats. Hence, supporting our notion that proton diffusion along the native BSA mat is aided by the formation of H-bonds between water molecules and the surface of the protein fibril, where in the chemically-modified mats, the surface is less accessible to water molecules. The more hydrophobic environment close to the protein surface of the modified mats, the less interaction between water molecules and the protein surface, hence, the more disruption of the H-bonds network. Our PC measurements (Fig. 1) also support our latter notion, and upon disruption of the H-bonds network, either by our chemical modifications or by increasing the aprotic solvent content, the conductivity across the mat is being reduced. Whereas, the mat with the most hydrophobic protein surface, the BSA-Hex mat, and the mat with the highest content of the aprotic solvent have the lowest measured PC. The same trend also appears in our calculated protonic mobility values (Table 1).

**Fig. 7:**
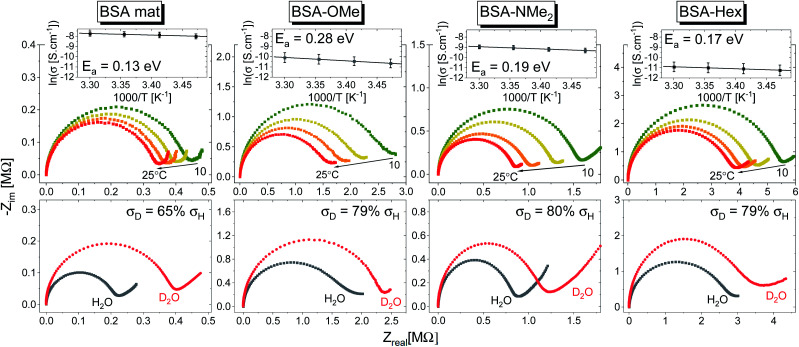
(Top) Temperature dependence studies of the PC across the various BSA mats. The insets show the Arrhenius plot of the logarithmic of conductivity (averaged of at least 3 replicas) as a function of inverse temperature along with the calculated activation energy (*E*_a_). (Bottom) Kinetic isotope effect of the PC across the various BSA mats upon deuterating the sample along with the calculated decrease in measured conductivity upon deuteration.

While trying to justify the different PC mechanism between the native BSA mat to the chemically-modified ones, or better to say different contributions of different PC mechanisms, we need to discuss PT on the molecular level. The PT efficiency between a proton donor to a proton acceptor is determined by the proton transition probability (*W*) that can be written in its general form:^29^
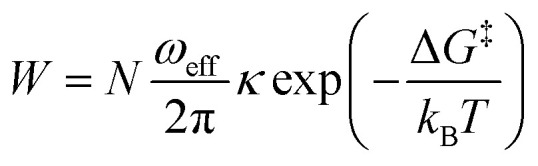
where *N* is the dimensionality of the PT, which is similar for all our samples, *ω*_eff_ is the effective vibrational frequency associated with the PT, *κ* is the quantum mechanical transmission coefficient of the PT event and Δ*G*^‡^ is the Gibbs activation free energy. Here we will refer to the latter as activation energy (*E*_a_) as entropic changes are most likely negligible compared to enthalpic ones. Different PC mechanisms will have different values for the different factors. Hence, to get further insight on the PC mechanism across the different BSA mats here, we conducted two further types of experiments that influence these factors (Fig. 7). The first experiment is temperature dependent study, which can result in a measured activation energy of the process (Fig. 7 top panels). The second experiment is observing the kinetic isotope effect (KIE) of the measured PC upon deuterating the sample (Fig. 7 bottom panels). Due to double the size of a deuteron compared to a proton, the KIE influences both the effective vibrational frequency as well as the transmission coefficient.^29^

In general, no matter what the PC mechanism is, it should be a thermally activated process, and indeed, we observed a temperature-dependent behaviour in our EIS measurements for all the different modified BSA mats (Fig. 7 top panels). In these measurements, we observed that the calculated activation energy (*E*_a_), by linear fitting the logarithms of the conductivity (*σ*) as function of inverse temperature (*T*) to Arrhenius equation, 
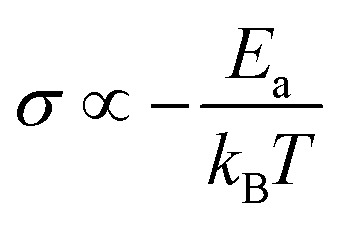
, altered between the different BSA mats, whereas all the modified BSA mats had higher activation energy compared to the unmodified mat. The relatively high activation energies of all the modified BSA mats suggest that the PC mechanism across the water channels of the modified mats (Scheme 2b) is not the vehicular mechanism, as it is expected to have lower activation energy (<0.09 eV) than we observed.^29^ The increase in activation energy upon the chemical modification suggests the involvement of an energy barrier for the proton hopping step between the different proton's mediators within the BSA mat, and that the mechanism is over-the-barrier type of mechanism.^30^ As can be observed in our schematic representation (Scheme 2), the chemical modifications result in a less dense hydrogen bonding network as there are less proton donors or acceptors in the system (also manifested in the lower carrier density, Table 1). According to the proton hopping mechanism,^29^ a dense hydrogen bonding network (as in the native unmodified BSA mat) should have an averaged lower difference between the equilibrium proton donor–acceptor distance to the one in the transition state, which should lead to a lower activation energy, as we observed in our results.

The second measurement that we performed was to follow the KIE upon deuterating our samples (Fig. 7, bottom panels). In general, a PC process involving water molecules should have a positive KIE, meaning a decrease in conductivity upon deuteration, as we observed for all our samples.^31^ According to the Grotthuss mechanism of proton hopping along a hydrogen bond network, the KIE should result in around 29% decrease in proton diffusion upon deuteration.^32^ All our measured KIE are around this value, hence, in line with our temperature dependence studies, it is a further justification that our measured PC is resulting from proton hopping both before and after the chemical modifications. As a comparison, the KIE of the vehicular mechanism should result in less than 10% decrease in proton diffusion upon deuteration.^29^ The KIE magnitude of proton hopping is resulting from effective vibrational frequencies (*ω*_eff_) associated with the proton/deuteron as well as differences in the potential surfaces of the proton hopping reaction.^29^ Since our system is highly complexed, it is rather impossible to resolve each proton hopping step along the hydrogen bonding network. Nevertheless, the similar change in the measured KIE for the different modified BSA mat compared to the native one suggests a similar change of the proton hopping characteristics, hence supporting our representation for the different PC mechanisms (Scheme 2).

All in all, our measurements here support our notion that PC across the native unmodified mat is along a dense hydrogen bonding network comprising the amino acids side groups and water molecules, whereas upon the various chemical modifications, this network is being disrupted, with less contribution of the amino acids side chains. Nevertheless, it is important to stress here that some of our observed changes (such as in the recently discussed activation energy and KIE) can be considered relatively minor (a change of ∼20–30% from the unmodified mat). Here, we should take into consideration that several functional groups along the protein backbone can participate in the hydrogen-bonding network. Hence, it is likely that the PC mechanism is not fundamentally different upon the chemical modifications, but it is rather a change in the ratio between proton hopping through water to the one involving protein's functional groups for the different chemical modifications and for the native mat.

## Conclusions

In summary, we demonstrate here our ability to modulate the PC across electrospun BSA mats together with its protonic mobility and proton carrier density using simple chemical modifications that target either oxo-amino acids (Asp or Glu) or free primary amines (Lys) within the mat. We show that both these amino acid groups contribute to the ability of the material to mediate protons, meaning that after the modification, the PC was hindered. We further show that making the surface of the protein more hydrophobic, using hexyl chain modification, resulted in further decrease in the PC. Our results indicate that the protein surface has a crucial role in the PC efficiency, which stems from the ability of the amino acid to participate in the hydrogen bonds network of surrounding water molecules. We have used the fluorescent Trp residue within the protein to have a close-up look at the local hydration layer surrounding the protein material together with its dynamicity for the different chemically modified BSA mats in comparison to BSA mats containing different amount of water molecules in aprotic solvent. Our steady-state and time-resolved fluorescence measurements reveal the ability of the protein surface to form H-bonds with surrounding water molecules, as well as a transient formation of a Trp radical that can undergo a proton-coupled electron transfer process. Our electrical and spectroscopic measurements allow us to have a better insight on the PC mechanism of the material for the different chemically modified mats, concerning proton hopping involving water molecules and protein's side groups *vs.* proton hopping primarily *via* water molecules. The use of proteins for the formation of proton conducting material is an emerging field. Our BSA-material are easy to form on the macroscale, and due to the low commercially cost of BSA they are highly affordable. Our ability to both modulate the PC across the material in an easy manner, together with our new insights on the local inner hydration environment and the PC mechanism, makes the BSA-based material a very attractive one, both as a platform to study long range PC across proteins, as well as to any future application of a proton conducting material.

## Experimental section

### Preparation of BSA mat

BSA mat was prepared by electrospinning. Bovine serum albumin (MP Biomedicals) was dissolved in 90% 1,1,1,2-tetrafluoroethane (TFE) (Apollo scientific) to a final BSA concentration of 14% (w/v). 5% (v/v) of β-mercaptoethanol (Alfa Aesar) was added to the solution. A custom built electrospinning system was used, where a bias of 12 kV was applied on a 24-gauge blunt needle, and the collector was grounded. The distance between the collector and the end of the needle was 12 cm, and the rate of injection was 1.3 mL min^−1^.

### Chemical modifications of the BSA mat – methyl ester modification (BSA-OMe)

The BSA mat was modified using Fischer–Speier esterification method.^33^ A piece of BSA mat (∼7–8 mg) was kept in methanol and 0.02 N HCl was added drop wise at room temperature. The solution was stirred for 24 h and the methanol was removed from the reaction vessel. *N*-methylation modification (BSA-NMe_2_): the BSA mat was modified using Hofmann exhaustive methylation method.^34^ A piece of BSA mat (∼7–8 mg) was kept in methanol and methyl iodide was added (in 1 : 2 molar ratio compared to BSA) and stirred for 24 h. *N*-hexylamide modification (BSA-Hex): *N*-hexylamine was coupled to carboxylic groups of the BSA mat using EDC/NHS coupling. A piece of BSA mat (∼7–8 mg) was kept in 5 mL water and stirred for 15 min. Then EDC HCl (0.1 mmol) and Sulfo-NHS (0.1 mmol) were added into solution and stirred for 1 h at room temperature. This is followed by addition of hexylamine (0.1 mmol) to the mixture and further stirring for 48 h. For all chemical modifications, to remove any impurities (mainly excess H+), the modified BSA mat was washed several times in methanol and water mixture until the pH of the washing solution reached to pH 7. There was not any observable change in morphological properties between the different modified BSA mats used here.

### Spectroscopy measurements

The FTIR spectra was measured using a Bruker Tensor 27 spectrometer equipped with an attenuated total reflectance (ATR). For each measurement background was recorded in air and subtracted from the spectra. The Raman spectra was recorded using Horiba Jobin Yvon (LabRAM HR Evolution®) spectrometer equipped with a 532 nm visible laser excitation source. Raman scattering was collected using am optical microscope. The steady-state fluorescence measurements were recorded using an FS5 (Edinburgh instruments) spectrofluorometer using an excitation wavelength of 280 nm. The fluorescence lifetimes were measured by time-correlated single-photon counting (TCSPC) technique, using CHIMERA spectrometer (Light Conversion) with an excitation wavelength of 280 nm. The laser system was based on a 10 W Yb-based laser amplifier (PHAROS, Light-Conversion) with pulses of <190 fs, operating at 1 MHz (pulse intensity of 10 μJ). The laser beam (at 1035 nm) was seeded into an optical parametric amplifier (ORPHEUS, Light-Conversion) followed by second and fourth harmonics generation. Here we prepared the sample by using a piece of BSA mat (1 cm × 1 cm) kept in ×0.2 PBS buffer, pH 7.4 for ∼2 h to adjust the inner pH of the mat to be neutral, followed by washing with water to remove any excess buffer. The wet mat was placed in vacuum chamber overnight for preparation of dry mat. The dry mat was placed between two quartzes plate and this configuration was used for the steady state and time resolved measurements in the reflection mode. To calculate the lifetime, the fluorescence decay curves were analyzed by Carpetview fitting program. The fluorescence decays have been recorded at 10 nm interims throughout the range (300–450 nm) of the fluorescence spectrum to build the time-resolved emission spectra (TRES). The fitted fluorescence decay has been scaled with a steady state spectrum to construct time-resolved area normalized spectra (TRANES).^35,36^ The solvent correlation function, *C*(*t*) was constructed using the following eqn:
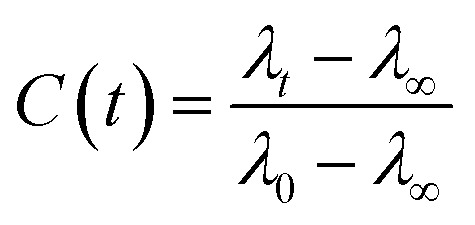
where *λ*_0_, *λ*_*t*_, and *λ*_∞_ are the emission maximum at time 0, *t* and ∞, respectively (*λ*_∞_ signifies the spectrum of the fluorophore after complete solvation).

### Electrical measurements

The impedance measurements were carried out using a MTZ-35 impedance/gain-phase analyzer (Bio-logic). The washed BSA mats as described above were placed onto gold finger electrode substrates (distance of 2.5 mm between gold fingers) and gently dried with filter paper to remove excess water not tightly bound to the surface. For the kinetic isotope measurements, the mats were vacuum dried and re-immersed in D_2_O. Micromanipulator probes were used to contact the gold electrodes. A frequency range of 10 MHz–10 Hz was used with applied ac bias of 50 mV (no dc bias was applied). The temperature dependent study was carried out using a Peltier containing probe station (INSTEC). Conductivity of mat is calculated using the following equation: 
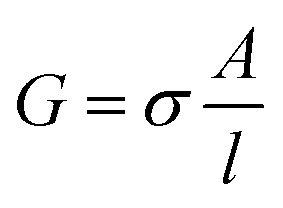
, where *G* is the conductance, *σ* is the conductivity, *A* is the cross-sectional area of mat (*A* = thickness of mat × wide of mat) and *l* is the distance between two electrodes. The thickness of measured BSA mat was ∼0.08 mm and dimension of the mats was around 1 cm × 1 cm. The three-terminal transistor devices were fabricated using heavily p-doped silicon wafers with SiO_2_ dielectric layer (110 nm). The substrate was cleaned using acetone, methanol, isopropanol and ethanol – sonicating in each separate solvent for 5 minutes and in given order. After ethanol sonication, substrates were washed with distilled water. 100 nm Au on-top of 10 nm Cr electrodes were deposited through a shadow mask using a thermal evaporator at a deposition rate of 2 Å s^−1^ under 5 × 10^−7^ Torr at room temperature for the source/drain electrodes of the transistors. Finally, BSA and modified BSA mats were placed on top of the patterned silicon wafer (distance of 100 μm between interdigitated gold electrodes) and gently dried with filter paper to remove excess water. The transistor measurement was acquired using two source measuring units (B2901A, Keysight), one for the source–drain and the other for the gate.

## Conflicts of interest

There are no conflicts to declare.

## Supplementary Material

SC-011-C9SC04392F-s001
