# Correction: Exploring long-range proton conduction, the conduction mechanism and inner hydration state of protein biopolymers

**DOI:** 10.1039/d0sc90113j

**Published:** 2020-06-04

**Authors:** Somen Mondal, Yuval Agam, Ramesh Nandi, Nadav Amdursky

**Affiliations:** Schulich Faculty of Chemistry, Technion – Israel Institute of Technology Haifa 3200003 Israel amdursky@technion.ac.il

## Abstract

Correction for ‘Exploring long-range proton conduction, the conduction mechanism and inner hydration state of protein biopolymers’ by Somen Mondal *et al.*, *Chem. Sci.*, 2020, **11**, 3547–3556, DOI: 10.1039/C9SC04392F.

The authors regret that in the original article there is an error in the equation presented in the sentence beginning “After calculating the mobility values, we estimated the charge carrier density…”. The equation should read as follows:*σ*_H^+^_ = *μ*_H^+^_ × *n*_H^+^_ × *e*

The Royal Society of Chemistry apologises for these errors and any consequent inconvenience to authors and readers.

