# Supplementary material for: Exploring long-range proton conduction, the conduction mechanism and inner hydration state of protein biopolymers
Source: Chem Sci. 2020 Mar 11;11(13):3547–56. doi: 10.1039/c9sc04392f (PMC8152808; doi:10.1039/c9sc04392f)
Supplement: SC-011-C9SC04392F-s001 [file SC-011-C9SC04392F-s001.pdf]

Electronic Supplementary Material (ESI) for Chemical Science.
This journal is © The Royal Society of Chemistry 2020

Supporting Information

Exploring Long-range Proton Conduction, the Conduction Mechanism and Inner Hydration State of Protein Biopolymers

*Somen Mondal, Yuval Agam, Ramesh Nandi and Nadav Amdursky**

Schulich Faculty of Chemistry, Technion – Israel Institute of Technology, Haifa 3200003, Israel.

**Corresponding Author: Nadav Amdursky, E-mail:* amdursky@technion.ac.il

FTIR Spectroscopy

The FTIR spectrum (Figure S1a) of BSA mats shows two intense peaks at ~1650 and 1540 cm^{-1}, correspond to amide-I (-C=O stretching) and amide-II (–C-N stretching and -N-H bending), respectively, whereas the peak at ~1395 and 3200 cm^{-1} were assigned to –COO^{-} side chain and -N-H bending of BSA mat, respectively.[1] Following the various modification we noticed the appearance and changings of several of the peaks. Upon methyl ester modification of the BSA mat (BSA-OMe), a new characteristic peak of ester appeared at 1735 cm^{-1} (Figure S1b).[2,3] Following the methyl esterification process, we observed that the amide-I is split in two separate band at 1630 and 1650 cm^{-1} which is due to the decomposition of amide-I.[4] Upon N-methylation (BSA-NMe$_2$) and hexylamine (BSA-Hex) modifications, we observed an increase in intensity of –C-H stretching at 2960 cm^{-1}, and another interesting increase in the –O-H stretching at around 3300 cm^{-1}. We also witnessed some small changes in the amide peak positions for all BSA modifications (Figure S1c), indicating some influence of the modification on the protein backbone.

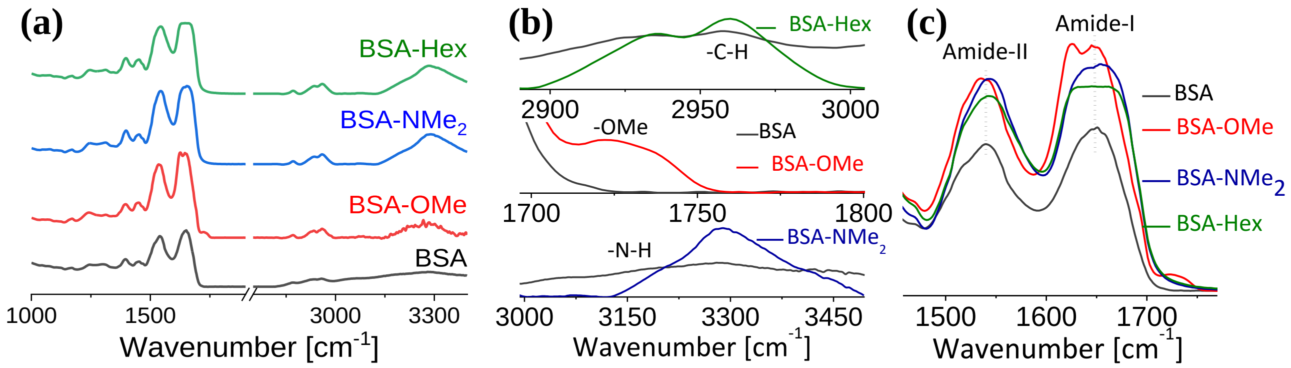

Figure S1. (a) FTIR spectra of the different chemically modified BSA mats along with the unmodified mat. (b and c) Zoom-in of (a) at different wavenumbers.

Raman spectroscopy

For the additional confirmation of modifications of BSA mat, we have carried out Raman spectroscopy which is very much useful to determine the protein structure and highly sensitive to the symmetrical vibrational modes of the aromatic side chains such as phenylalanine (Phe), tyrosine (Tyr) and tryptophan (Trp). Since our modifications here are not Raman active, the observed changes in the Raman spectrum are not big (Figure S2a), hence the above discussed FTIR characterization is superior in observing our modifications. Nevertheless, we can still see some difference between the different modified BSA mats. Here we have focused on the sharp peak of aromatic side chain (1000 cm^{-1}, Figure S2b) and a broad peak of amide I bond (1658 cm^{-1}, Figure S2c). A pronounced blue shift is observed in aromatic side chain stretching when modification is done with small methyl group but for long chain (Hex) modification a red shift is observed which is due to change of polarity around the aromatic side chain. In the amide I band position, we also observed a shift after the modifications, probably due to some minor change of structure. Overall, our Raman measurement indicates that the modification changed the environment, i.e., the polarity, within the BSA mats, and confirms the functional group modifications. We should further stress here that fluorescence spectroscopy is a much sensitive tool for following the changes in the environment and hydration layer within the BSA mats in comparison to Raman measurements (as well as FTIR ones), as detailed along the main text of the paper.

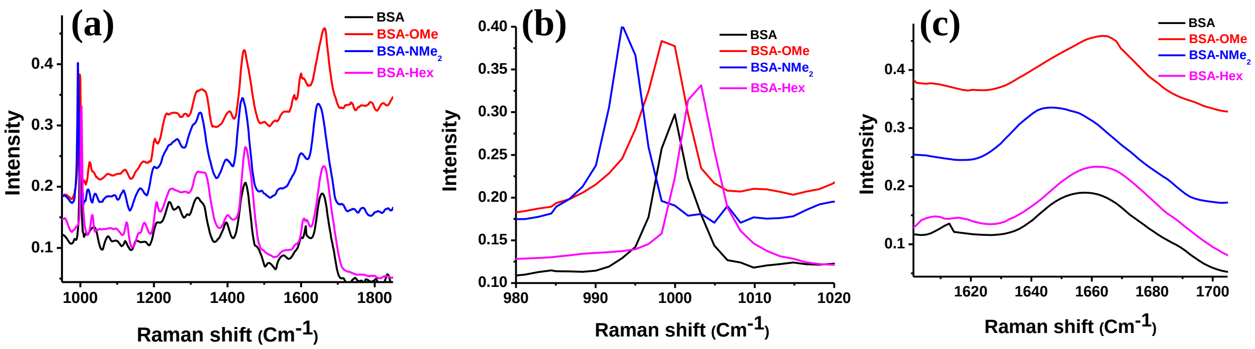

Figure S2. (a) Raman spectra of the different chemically modified BSA mats along with the unmodified mat. (b) Zoom-in of (a) at 980-1020 cm^{-1} (aromatic side chain). (c) Zoom-in of (a) at 1610-1700 cm^{-1} (amide I bond).

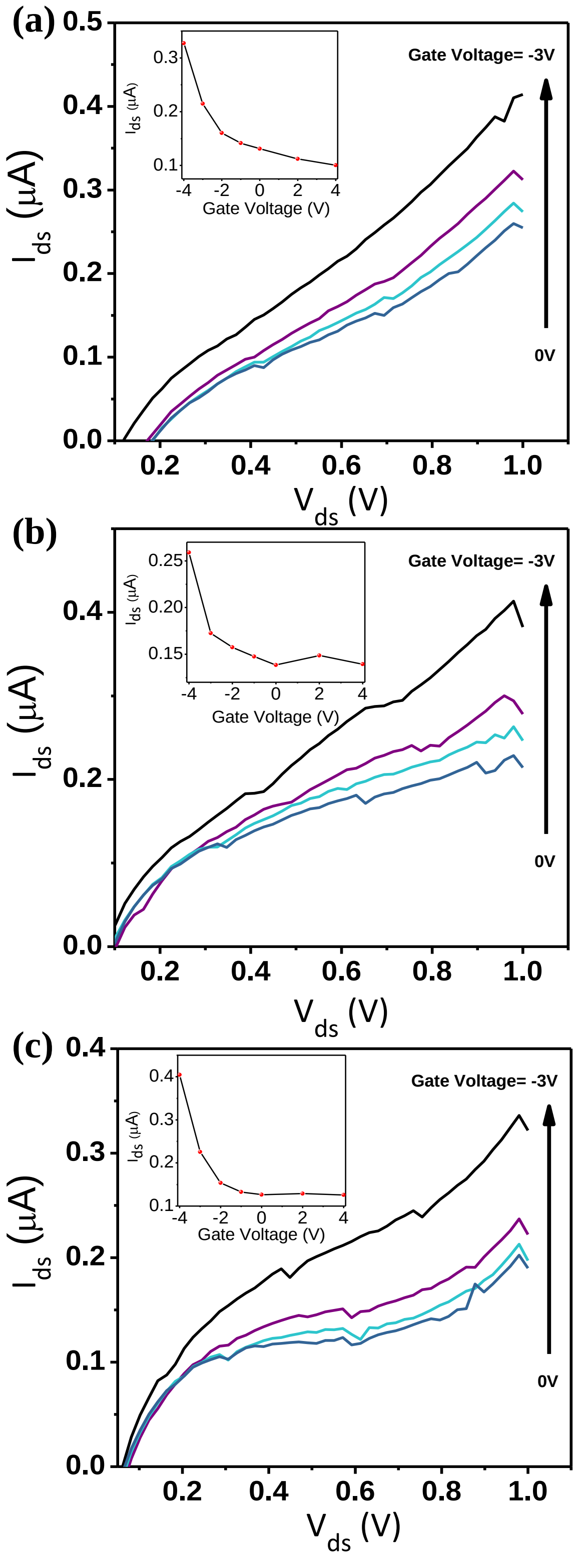

Figure S3: Typical H^+-FET measurements, source–drain current (I_{DS}) as a function of the source–drain voltage (V_{DS}) at different values of V_{GS} for (a) BSA-OMe (b) BSA-NMe_2 (c) BSA-Hex. The Insets show the transfer line characteristics of the device.

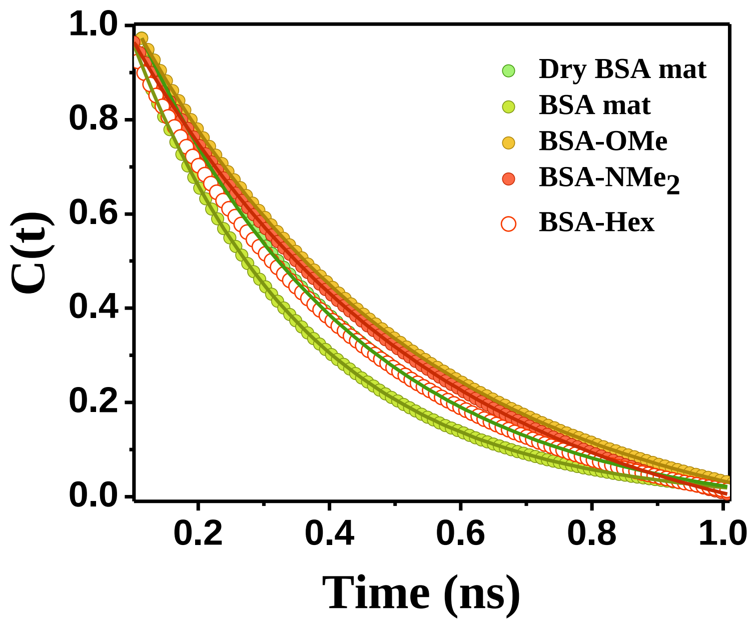

Figure S4: The constructed decay of solvent response function C(t) of Trp in different modified BSA mats.

References

1 S. H. Schneider and S. G. Boxer, *J. Phys. Chem. B*, 2016, **120**, 9672–9684.

2 I. M. Pazos, A. Ghosh, M. J. Tucker and F. Gai, *Angew. Chemie - Int. Ed.*, 2014, **53**, 6080–6084.

3 I. M. Tang, N. Krishnamra, N. Charoenphandhu, R. Hoonsawat and W. Pon-On, *Nanoscale Res. Lett.*, 2012, **7**, 1–10.

4. J. Grdadolnik, Y. Maréchal, *Biopolymers*, 2001, **62**, 40-53.
